# Associations of Perfluoroalkyl Substances with Prevalence of Metabolic Syndrome in Highly Exposed Young Adult Community Residents—A Cross-Sectional Study in Veneto Region, Italy

**DOI:** 10.3390/ijerph18031194

**Published:** 2021-01-29

**Authors:** Maryam Zare Jeddi, Teresa Dalla Zuanna, Giulia Barbieri, Aline S. C. Fabricio, Francesca Daprà, Tony Fletcher, Francesca Russo, Gisella Pitter, Cristina Canova

**Affiliations:** 1 Unit of Biostatistics, Epidemiology and Public Health, Department of Cardio-Thoraco-Vascular Sciences and Public Health, 35131 Padova, Italy; maryam.zarejeddi@unipd.it (M.Z.J.); teresa.dallazuanna@studenti.unipd.it (T.D.Z.); giulia.barbieri.1@unipd.it (G.B.); 2 Veneto Institute of Oncology IOV-IRCCS, 35128 Padua, Italy; aline.fabricio@aulss3.veneto.it; 3 Laboratory Department-Regional Agency for Environmental Prevention and Protection-Veneto Region, 37135 Verona, Italy; francesca.dapra@arpa.veneto.it; 4 London School of Hygiene and Tropical Medicine, London WC1H 9SH, UK; tony.fletcher@lshtm.ac.uk; 5 Directorate of Prevention, Food Safety, and Veterinary Public Health-Veneto Region, 30123 Venice, Italy; francesca.russo@regione.veneto.it; 6 Screening and Health Impact Assessment Unit, Azienda Zero-Veneto Region, 35131 Padova, Italy; gisella.pitter@azero.veneto.it

**Keywords:** epidemiology, PFAS, biomonitoring, metabolic syndrome, cardiovascular risk factors, metabolically healthy obesity

## Abstract

Background: Studies on the association between perfluoroalkyl substances (PFAS) and metabolic syndrome (MetS) are limited, and results are inconsistent. We aimed to examine the associations between PFAS serum levels and the prevalence of MetS among highly exposed young adults (ages 20–39) residents of a large area of the Veneto Region (North-Eastern Italy) primarily stemming from PFAS water contamination before September 2013. A total of 15,876 eligible young adult residents living in the investigated municipalities were enrolled in the study from January 2017 to July 2019. Methods: MetS was defined by using a modified harmonized definition requiring the presence of 3 of the following: obesity (body mass index ≥30), elevated triglyceride (TG), reduced high-density lipoprotein cholesterol, elevated blood pressure, and hemoglobin A1c ≥ 6.1% or self-reported diabetes mellitus or drug treatment for hyperglycemia. Multivariable generalized additive models were performed to identify the associations between four serum PFAS, including perfluorooctane sulfonic acid (PFOS), perfluorooctanoic acid (PFOA), perfluorohexane sulfonic acid (PFHxS), and perfluorononanoic acid (PFNA), and risk of MetS controlling for potential confounders. Results: A total of 1282 participants (8.1%) met the criteria of MetS with a higher prevalence among men. PFOA, PFHxS, and PFNA were not associated with the risk of MetS, whereas PFOS showed a consistent protective effect against the risk of MetS (OR 0.76, (95% CI: 0.69, 0.85) per ln-PFOS). However, we found statistically significant positive associations between PFAS serum levels and individual components of MetS, mainly elevated blood pressure and elevated TG. Conclusion: Our results did not support a consistent association between PFAS and MetS and conflicting findings were observed for individual components of MetS.

## 1. Introduction

Perfluoroalkyl and polyfluoroalkyl substances (PFAS) are a group of more than 4700 man-made chemicals containing an aliphatic fluorinated carbon chain [1]. Due to their unique chemical and physical properties, including oil and water repellence, temperature and chemical resistance, and surfactant properties, since the 1940s they have been and still are used in numerous industrial and consumer applications [2,3]. Evidence of internal PFAS exposure in humans is known and has been underlined in several national human biomonitoring studies conducted worldwide [4,5,6,7,8]. 

The general population is primarily exposed to PFAS through food, water, and dust exposure (ingestion and inhalation) [7,8,9]. However, exposures at levels higher than the general population have occurred around industrial production, manufacturing, and application sites mainly due to contaminated drinking water in communities in Italy, Sweden, Belgium, and the Netherlands [10,11], and around airports and military bases in Germany, Sweden, and the United Kingdom, as well as outside the EU [12].

Over the past decades, several lines of evidence have implicated an association between exposure to PFAS and higher serum cholesterol concentrations, hypertension, thyroid hormones, liver enzymes, and inconsistent results on perturbation of insulin and glucose homeostasis, markers of immunologic function, asthma, and children’s behavior [13,14]. However, reported associations with metabolic syndrome (MetS) have so far been inconclusive, with conflicting findings reporting null [15,16,17,18], adverse [19,20], and even protective associations [21].

MetS, also known as Insulin Resistance Syndrome (IRS) and Syndrome X, is a cluster of metabolic and anthropometric traits, which occur together more often than by chance alone, including abdominal obesity, hypertension, dyslipidemia, and hyperglycemia. MetS constitutes a powerful risk factor complex to identify individuals at increased risk for future Type 2 diabetes mellitus and cardiovascular disease (CVD) that has reached a large and growing world dimension [22]. 

Therefore, the main objective of the present study is to (1) describe the prevalence of MetS and its components in a highly exposed community and (2) to examine the extent to which PFAS serum concentrations are associated with the risk of MetS. The population included residents of the surrounding community of a PFAS manufacturing plant who were exposed for decades to drinking water contaminated by PFAS in Veneto region, Italy.

## 2. Materials and Methods

### 2.1. Participants 

The study population was recruited from an ongoing population-based health surveillance program approved with the Regional Government Deliberation n. 2133 on 23 December 2016. The health surveillance comprises individuals from more than twenty municipalities with PFAS-contaminated waterworks, so called “Red area” in Veneto region, Italy, due to the activities of a local manufacturing plant that started PFAS production since the late-1960s [11]. Clean water was provided in the Red area from September 2013. To be eligible, participants were required to live in the Red area, and be willing to follow to the health surveillance program. Information on this ongoing health surveillance program including study design, survey administration, participants enrollment as well as characteristics and a map of the Red area have been previously described in details [11]. Overall, 16,224 young adult residents, born between 1978 and 1999 (aged 20–39 years at the recruitment) and living in the investigated municipalities, were enrolled in the surveillance plan from January 2017 to July 2019. Participation rate for this population was 61%. Pregnant participants (*n* = 327) and individuals with missing information on the metabolic syndrome components (approximately 0.1% of the studied population, *n* = 21) were excluded leaving a total of 15,876 participants included in the statistical analysis.

A structured software-based questionnaire was administered collecting information on socio-demographics, personal characteristics, anthropometry, and an abbreviated health history at the enrolment.

Blood pressure was measured during the medical exam at the time of enrolment according to the European Society of Hypertension recommendations [23]. Non-fasting blood samples were collected from participants and shipped for biochemistry markers testing related to MetS at three laboratories within the Red Area (Arzignano, San Bonifacio, Legnago). In the present study, laboratory data included triglycerides (TG), high density lipoprotein cholesterol (HDL-C), and glycated hemoglobin (HbA1c) from whole blood. Blood sample collection and processing were described previously [11].

### 2.2. MetS Definition 

We adopted the definition of MetS from the Joint Interim Statement on MetS definition (JIS) [24]. The JIS definition is not hierarchical and does not require demonstration of any specific criteria such as insulin resistance or obesity per se; that is, every combination of three or more of the following criteria fulfils the diagnosis of MetS [24]: elevated waist circumference (population- and country-specific definitions); elevated TG: ≥150 mg/dL or drug treatment for elevated triglycerides; reduced HDL-C: <40 mg/L (male) or <50 mg/L (female), or drug treatment for reduced HDL-C; hypertension ≥130 mmHg systolic blood pressure (SBP) and/or ≥85 mmHg diastolic blood pressure (DBP) or antihypertensive drug treatment or self-declared hypertension; and elevated fasting glucose or drug treatment of elevated glucose. 

Waist circumference was not measured in the present study therefore body mass index (BMI) was used instead. AHA/NHLBI recommended Waist circumference cut points of ≥102 for men and ≥88 cm for women for people of European origin. The latter values are corresponding to the definitions of abdominal obesity (BMI  ≥  30 kg/m^2^) recommended by the World Health Organization (WHO), American Association of Endocrinology (AAE), and US National Program for Cholesterol Education (NCEP ATP III) [25]. 

In the absence of information on fasting blood glucose, HbA1c ≥ 6.1% (≥43 mmol/mol; as a screening tool for detection of type 2 diabetes) [26] or self-reported diabetes or drug treatment of elevated glucose (hypoglycemic medicines) were used as alternate indicators. Due to using non-fasting measurements, the cut-off value for elevated TG was set at 175 mg/dL [27]. Drug treatment for elevated TG and reduced HDL-C was limited to fibrates and statin, respectively.

Furthermore, metabolically healthy obesity (MHO) was established when obese subjects had none of the MetS components, and had no previous diagnosis of cardiovascular disease (self-reported history of acute myocardial infarction, stroke, angina pectoris, or cardiovascular intervention) [28].

### 2.3. PFAS Measurements in Serum

Twelve PFAS, including perfluorooctane sulfonic acid (PFOS), perfluorooctanoic acid (PFOA), perfluorohexane sulfonic acid (PFHxS),, perfluorononanoic acid (PFNA), perfluoroheptanoic acid (PFHpA), perfluorobutanesulfonic acid (PFBS), perfluorohexanoic acid (PFHxA), perfluorobutanoic acid (PFBA), perfluoropentanoic acid (PFPeA), perfluorodecanoic acid (PFDA), perfluoroundecanoic acid (PFUnA), and perfluorododecanoic acid (PFDoA) were quantified using HPLC MS/MS (Shimadzu UFLC XR 20 Prominence coupled to Sciex API 4000). The serum PFAS measurement method was previously reported [11]. The analytical method has been validated according to the UNI CEI EN ISO/IEC 17,025 regulation and the international guidelines on this topic. The measuring range of the method was 0.5–500 ng/mL. The repeatability of the method given as RSD of determined concentrations was below 10% for all measured PFAS at different levels and the accuracy was between 80% and 120%. The limit of detection (LOD) and limit of quantification (LOQ) values for 12 PFAS are presented in Supplementary Table S1. The concentration of serum PFAS was replaced with the value of LOQ divided by the square root of 2 if the concentration was inferior to LOQ. We excluded serum PFAS with detection rate <40% (8 PFAS) in current statistical analysis. Four PFAS were retained in subsequent analyses and their detection frequencies were PFOS: 99.7%, PFOA: 99.86%, PFHxS: 96.72%, and PFNA:49.86%. Participants with the most extreme outliers on PFAS exposure including PFOA > 700 mg/L (*n* = 6), PFOS > 50 mg/L (*n* = 15), PFHxS > 100 mg/L (*n* = 3), PFNA >10 mg/L (*n* = 1) were excluded from the analyses.

### 2.4. Statistical Analysis

Demographic characteristics were presented using frequencies and proportions for categorical variables and the mean ± standard deviation (SD) for continuous variables among study population classified according to MetS status stratified by gender. Each characteristic of the participants was compared between MetS and non-MetS by using t-test for continuous variables or chi-square test for categorical variables. In order to adjust the skewed distributions, serum PFAS levels were natural log transformed (ln-PFAS). We also analyzed the Spearman correlation between serum PFAS concentrations.

The shape of possible dose-response associations between continuous PFAS serum levels and MetS prevalence was modelled through thin plate spline smooth terms for the exposures and continuous covariates. Degree of smoothing was selected by Generalized Cross Validation as implemented in the R package mgcv [29]. Since the spline analysis showed associations compatible with a linear relationship on the natural-log transformed PFAS, linear regression coefficient (β) and 95% confidence intervals (CI) were reported. The PFAS quartiles (IQ, IIQ, IIIQ, IVQ), were examined in relation to MetS as a dichotomous outcome with the first PFAS quartile as the reference, and a *p*-value for trend across the quartiles was calculated. 

We estimated the odds ratios (ORs) of MetS using Multivariable Generalized Additive Models (GAMs) with a binomial link function to identify associations between serum PFAS levels and MetS (yes or no for having at least 3 components of MetS) and the presence of MetS components (yes/no for a given component), controlling for several sociodemographic covariates.

Potential confounders were determined a priori based on associations between PFAS and metabolic outcomes from previous studies [17,30,31]. Thereafter a Directed Acyclic Graph (DAG) was used (Supplementary Figure S1) to identify set of a minimally sufficient set of variables to control for confounding.

The final model for adjustment was Model 1 including gender, age, country of birth, smoking status (current smoker/former smoker/never smoked), diet (meat, fish, cheese, eggs, fruits and vegetables, sweets, snacks and sugary drinks, salt intake), alcohol consumption (categorized in 0, 1–2, 3–6, 7+ alcohol units per week), time-lag between enrollment and the beginning of the study, number of deliveries, clinical centers where the blood pressure have been measured and the questionnaire have been filled, education and physical activity (light/moderate/heavy). Model 2 was performed only for individual components of MetS, including all covariates of model 1 plus all other components of MetS.

Associations described in the results as statistically significant use the criterion *p* < 0.05. 

The statistical software STATA/SE version 13.0 (Stata Corp LP, College Station, TX, USA) and R (R Development Core Team 2010, R Foundation for Statistical Computing, Vienna, Austria. ISBN 3-900051-07-0, URL: http://www.R-project.org/) were used for statistical analyses. 

### 2.5. Sensitivity Analyses

We assessed the robustness of the results in various sensitivity analyses. A sensitivity analysis was conducted to consider estimated glomerular filtration rate (eGFR) as an additional covariate in the analyses since eGFR value < 90 mL/min/1.73 m^2^ has been associated with increased risk of metabolic diseases, obesity, hypertension, type 2 diabetes, CVD, and non-alcoholic fatty liver disease (NAFLD) [32,33]. 

Furthermore, a sensitivity analyses was performed defining MetS by International Diabetes Federation (IDF) criteria [34]. The major difference between these two definitions is that the IDF criteria regards central obesity as an essential and core criterion of MetS with cut point of waist circumference (WC) ≥94 cm for men and ≥80 cm for women in Europeans which corresponds to BMI ≥25 kg/m^2^ (“overweight”). 

Lastly, a sensitivity analyses was carried out restricting our population to nonsmokers because unhealthy lifestyle, such as smoking is linked to higher prevalence of MetS [35].

## 3. Results

### 3.1. Population Characteristics and MetS Components

The study population consisted of 15,876 individuals that were almost evenly distributed by gender (48.61% men, and 51.39% women) with a mean age of 30.0 years (SD 5.8 for both). The overall prevalence of the MetS was 4.68% (*n* = 743) based on the JIS definition, with a significant higher prevalence in men than women (Table 1).

The most commonly reported components were elevated blood pressure (33.9%) followed by reduced HDL-C (15.2%). The mean level of triglycerides was 104.9 ± 73.6 mg/dL and elevated triglycerides (≥175 mg/dL) was shown in 11.48% of participants. 

When stratified by gender, more men than women met MetS criteria for almost all the components of MetS, particularly for elevated TG, elevated blood pressure and obesity. In contrast, more women showed reduced level of HDL-C (Table 1). The median HDL-C level was 58.64 mg/dL, indicating that 16.5% of women were below the cut off for reduced HDL-C (<50.0 mg/dL) (Table 1).

However, among obese population (BMI ≥ 30: *n* = 1383, 8.71%), a total of 303 subjects (21.91%) did not have any metabolic abnormalities, as well as no previous diagnosis of cardiovascular disease, as defined by the MHO phenotype. The prevalence of individuals with MHO was found higher in women (*n* = 199, 29.05%) than in men (*n* = 104, 14.90%) (Supplementary Table S2). 

Significant differences in demographic and lifestyle characteristics according to MetS status were observed among participants (Table 2). Compared to the non-MetS group, the MetS group had significantly higher proportions of current and former smoking status, lower level of education (elementary–middle school), higher meat and salt consumption, lower fruits and vegetables consumption, and origin from HMPC countries. The proportion of not drinkers as well as heavy drinkers (more than seven alcoholic units per week) was significantly higher in the MetS group than in the non-MetS group. The prevalence of women with two or more deliveries was significantly higher in the MetS group (Table 2). 

Serum PFAS concentrations of both MetS and non-MetS participants is presented in Table 2. The concentrations of PFOA and PFHxS were higher among people with MetS than those with non-MetS. There was almost no difference of serum PFOS and PFNA between MetS and non-MetS participants. The geometric mean (range) of serum concentrations were 67.66 (0.70, 1400.0) µg/L for PFOA, 7.54 (<LOQ, 127.0) µg/L for PFHxS, 4.54 (<LOQ, 142.0) µg/L for PFOS, and 0.58 (<LOQ, 39.7) µg/L for PFNA, in the MetS group (Supplementary Table S4). These measures among the non-MetS group were 59.37 (<LOQ, 1253.3) µg/L for PFOA, 5.89 (<LOQ, 77.1) µg/L for PFHxS, 4.63 (<LOQ, 124.0) µg/L for PFOS, and 0.53 (<LOQ, 8.8) µg/L for PFNA. Men had 2–3-fold higher serum PFAS concentrations compared to women (Supplementary Table S3). Additionally, Spearman’s rank correlation analysis showed that all PFAS compounds were moderately to strongly correlated with one another (*p* < 0.05). The most highly correlated compounds are PFHxS with PFOA, the two PFAS showing highest average concentrations (rs = 0.907); the least correlated are PFNA with PFOA (rs = 0.397). The Spearman correlation coefficients of PFOS with PFOA and PFHxS were 0.634 and 0.680, respectively; and the correlation coefficients of PFNA with PFHxS was 0.403. 

### 3.2. Association between PFAS and MetS and Its Components 

Table 3 and Table 4 display the prediction of serum PFAS on odds ratios (ORs) with 95% confidence interval (CI) for MetS and its components as binary outcomes per natural-log unit (ng/mL) increase in levels of PFAS as well as in categorical (based on quartiles of exposure). As shown in Table 2, the odds of MetS were not significantly associated with any of PFOA, PFHxS and PFNA concentrations. Serum ln-PFOS concentrations were significantly associated with a lower prevalence of MetS (OR 0.76, (95% CI: 0.69, 0.85) per ln-PFOS) in covariate-adjusted models. When stratified by gender, the effect estimates were similar (Table 3). The tests for trend by treating PFAS level in quartiles showed only a significant trend for PFOS. The OR for the highest compared with the lowest quartile of PFOS was 0.55 (95% CI: 0.43–0.70, *p*-trend < 0.01). The negative association between PFOS concentrations and the risk of MetS remained consistent with increasing PFOS quartiles and these associations were more pronounced in women. 

The associations between the serum PFAS levels and individual components of the MetS are summarized in Table 4. After adjustment by other components of the MetS in addition to other cofounders, increased serum PFAS concentrations were associated with a higher prevalence of hypertension (PFOA OR 1.05 (95% CI 1.01–1.08); PFOS OR 1.10 (95% CI 1.03–1.17); and PFHxS OR 1.07 (95% CI 1.03–1.12)). Increased serum PFOA and PFHxS concentrations were also correlated with a higher prevalence of elevated triglyceride as defined in MetS criteria (PFOA OR 1.10 (95% CI 1.05–1.16)); and PFHxS OR 1.09 (95% CI 1.02–1.16)). PFNA was positively but non-significantly associated with increased risk of hypertension (OR 1.10 (95% CI: 0.99, 1.21)) and with higher risk of higher triglyceride level above the MetS criteria (OR = 1.06 (95% CI: 0.92, 1.23)). 

In contrast, increased serum levels of PFOA, PFOS, PFHxS, and PFNA were significantly associated with decreased risk of other components, with the most consistent pattern seen for PFOS (Table 4). 

Sensitivity analysis were conducted to assess the constancy of the association between investigated PFAS and the prevalence of MetS while adding eGFR in the models as possible confounder. Adjusting for eGFR did not change the results for PFOS, PFOA and PFHxS, whereas, the null association turn out to be significant between PFNA and the prevalence of MetS. 

Considering the IDF definition, about 4.7% (*n* = 743) of the participants met the IDF criteria for MetS, with higher prevalence in men (6.6%) than women (2.9%). Overall, 3.55% (based upon JIS definition) and 6.08% (based upon IDF definition) of the survey participants had at least three of the MetS components present (Supplementary Table S4), whereas less than 0.1% met the criteria for all five components. In the total study population, 668 (4.21%) of the participants had met the criteria of MetS by both JIS and IDF definitions, with rates of 5.75% and 2.75% among men and women, respectively. 

The association between PFAS and the risk of MetS remains consistent in the models while using the IDF definition. Serum PFOS concentrations were significantly associated with a lower risk of MetS defined by the IDF criteria with OR = 0.70, (95% CI: 0.61, 0.79) per ln-PFOS. Modelling PFAS by quartiles yielded consistent results.

Moreover, the results of the sensitivity analysis restricted to non-smokers were consistent with the main result that none of the PFAS was positively associated with MetS and the negative associations between serum PFOS were robust.

## 4. Discussion

To our knowledge, this is the first study investigating the association between PFAS exposure and MetS in a highly exposed community of residents in Europe. 

The observed prevalence of MetS was 8.1% among young adult population aged at 20 to 39 years old, comorbidities conveying high risk of both cardiovascular disease and type 2 diabetes. 

However, PFOA, PFHxS, and PFNA failed to show positive significant associations with MetS and depending upon the specific PFAS and MetS component under investigation, either null, deleterious or protective associations were observed with borderline to statistical significance. Further, multiple models demonstrated that PFOS was associated with decreased risk of MetS and consistently for all but one of its components (hypertension). For individual components of MetS, all the investigated PFAS were positively associated with hypertension but with decreased risk of reduced HDL-C.

In other epidemiological studies, inconsistent findings exist regarding the association between PFAS and MetS. Overall, no association was observed between PFOA, PFOS, PFHxS, and MetS in different cross-sectional studies, which is concordant with our analysis [15,16,17,18,21,30,36]. Only two studies one on a small scale (*n* = 148) of Chinese adult men (19–60 years old) and the other of 1501 adults from a cross-sectional study, the “Isomers of C8 Health Project in China” found a significant association between linear PFOA and the risk of MetS with ORs of 29.4 (95% CI, 2.90–299.7), after adjustment only for age [19] and 1.99 (95% CI, 1.40, 2.83) after adjustment for age, sex, income status, smoking status, alcohol consumption, regular exercise, seafood consumption, and eGFR [20]. A protective (negative) significant association between PFOS concentrations and the risk of MetS is only reported in the present study and the study by Leary et al. (2018), but in the latter the association disappeared after adjusting for confounders [16]. In the study conducted by Ye et al. (2020) an isomeric-specific association between PFOS and MetS was found, in which was statistical significance for branched PFOS isomers but null for linear PFOS [20]. Different structure of perfluoroalkyl branched-chain between linear and branched PFAS may influence their pharmacokinetic characteristics (e.g., binding affinity to protein, elimination half-lives) and their toxicity. For PFNA, results are inconsistent across the studies. Recently, Christensen et al. examined the data from the National Health and Nutrition Examination Survey (NHANES) 2007–2014 and reported that the prevalence of MetS defined by JIS definition was 37% among U.S. adult population (≥20 years old) and only PFNA was associated with an increased risk of MetS, when controlling for multiple PFAS [21]. Likewise, a cross-sectional study on 122 adult participants (44–56 years old) of the Croatian island on 2007–2008 identified a 2–3 times increased risk of MetS as defined by the ATP III criteria for a natural log unit increase of PFNA [30]. In the cross-sectional study conducted by Yang et al. PFNA serum levels above the median were associated with 10.9–fold (95% CI, 2.00–59.1) increased risks of MetS, while after adjustment for age, the association was not robust [19]. In the present study, a significant positive association was found between PFNA and MetS after extra adjustment for eGFR. In contrast, increased serum PFNA concentrations were associated with a lower prevalence of the MetS according to NHANES 1999–2000 and 2003–2004 data and borderline but not significant based on subsequent analysis of NHANES 2013–2014 data [16,36]. The PFAS quantified at the highest serum concentration in this population was PFOA followed by PFHxS. For PFOA, the median was 35.9 μg/L, which is about 12–32 times higher than other European studies with background exposure [30,31] as well as Canadian and U.S. general populations [15,16,17,21,36], even 4–5 years after eliminating the exposure via contaminated drinking water in the Veneto region. Overall, the PFAS concentrations found in total serum in the young adult population was around five times higher for PFOA and PFHxS than the average burden reported for Europe 7.7 µg/mL and 0.67 µg/mL, respectively [9]. In contrast, the PFOS median concentration (3.7 μg/L) was significantly lower in our study population than the other studies [18,20] but in a roughly similar range to the median PFOS concentration of a Chinese adult population (3 μg/L) [19]. Nevertheless, the medians of serum PFOS concentration here were almost two times higher than the median concentrations in serum/plasma in the general adult population in Europe (1.9 µg/mL) [9]. In general, the observed pattern of PFAS levels, relating among participants defined by MetS criteria, were comparable with the results of the four cross-sectional studies. Overall, no association was observed between PFOA, PFOS, PFHxS, and MetS in different cross-sectional studies, which is concordant with our analysis [17,18,19,20]. For PFNA, the level in this study was relatively lower than the other studies. Nevertheless, concentrations alone do not explain the discrepancy in findings between the different studies. Moreover, serum PFAS concentrations were strongly correlated with each other in all the studies and therefore, effects of specific PFAS cannot be distinguished.

For individual metabolic components, the literature is also not entirely consistent. Hepatic steatosis effects supported by convergent data from both the toxicology and epidemiology studies might be considered as a suggested contributing mechanism to the altered cholesterol following by PFAS exposure [9,33]. This association is consistently evident for altered total and LDL cholesterol but not for HDL-C and TG. Our findings of inverse associations between PFAS, triglycerides (mainly for PFOS) and HDL-C are in the same direction as other studies [17,31,36,37]. As for elevated blood pressure, positive associations are reported for some PFAS in recent studies [11,18,38]. 

To date, few studies have investigated the association between PFAS and overweight/obesity with inconsistent results [39,40,41]. A recent cross-sectional study among 1612 Chinese adults (1204 men and 408 women), ages 22–96 years old, indicated that increased serum concentrations of PFOS, PFOA, and PFNA were positively associated with overweight/obesity and PFNA and PFOA with increased WC [42]. A significant association was also observed between PFNA and BMI ≥ 25 kg/m^2^ but not remarkable association with obesity (BMI ≥ 30 kg/m^2^) in Croatian island adult population at ages 44–56 years [30]. Statistically significant positive associations with BMI were reported for PFNA with serum concentrations above the medians in the study of Chinese men aged 19–60, after adjustment for age [19]. As for our data, the observed PFAS–BMI associations were mainly negative and for some PFAS null and therefore considered inconclusive. Among investigated community residents, there were a total of 5204 overweight and obese (BMI ≥ 25: 32.8%) participants with higher prevalence in men (40%) than women (26%), among which 1685 participants (32.38%) did not meet any of the criteria for MetS and did not have previous diagnosis of cardiovascular disease, so called MHO. The prevalence of MHO was higher in overweight and obese (BMI ≥ 25) women (*n* = 866, 41.40%) than in men (*n* = 819, 26.32%) (Supplementary Table S2). Whereas, considering overweight and obese based on IDF criteria for MetS did not alter the association between PFAS and MetS.

Although the directions of our relationships with MetS are consistent with most of other studies, it is notable that our study population was restricted to the age group between 20 and 39, while the most frequently investigated age group in other studies is that over 20 years old. According to the literature, aging contributes to increase prevalence of MetS since many predisposing conditions, such as hypertension, obesity, and insulin resistance increase in prevalence during aging. However, we do not know whether results may change considering older community residents. 

Altogether, clear conclusions cannot be drawn on PFAS and MetS from our study and previous literature, due to the inconsistency in the results between different studies and the cross-sectional nature of the studies. Conflicting observations mostly are attributable to differences in cut-off points for criteria used for defining MetS associated with each PFAS, PFAS concentrations (occupational vs. community vs. environmental exposures), study design (cross-sectional vs. longitudinal studies) and age and gender-specific differences. In addition, any failure to fully adjust for potential confounders could be an alternative explanation for the conflicting results observed in human studies.

Overall, MetS is a cluster of interrelated risk factors (obesity, insulin resistance, dyslipidemia, and hypertension) all known as risk factors of cardiovascular diseases (CVD), therefore different mode of action/adverse outcome pathways (AOPs) of PFAS could influence the final results [14,43]. For instance, the PFAS-to-lipid associations are explained in animal studies by transactivation of peroxisome proliferator-activated receptor α (PPARα) contributing to a liver metabolic pathway which plays an important role in the regulation of cholesterol and triglyceride levels; or oxidative stress has been proposed to be a key player in the pathogenesis of hypertension [44,45,46,47]. Other putative molecular initiating/key events for PFAS might be explained by mitochondrial dysfunction, interference of protein binding, partitioning into lipid bilayers (enterohepatic cycling of both bile acids and PFAS), altered calcium homeostasis, and inappropriate activation of molecular signals controlling cell functions [14,48]. As for CVD, few studies of occupationally exposed workers and non-occupationally exposed population have reported associations between PFAS exposure and CVD [49]. However, the potential mechanisms beyond the potential association are still not clear and several hypotheses have been made. The results of a study investigating alteration of microRNA expression indicated that modulation of miRNA expression may be a mechanism of toxicity of PFAS and in silico functional analyses suggested potential links between PFAS concentrations in serum, miRNA expression, specific miRNA target genes and perhaps onset of health effects such as cardiovascular [50]. Moreover, a recent study on the role of impaired platelet aggregation in increased cardiovascular risk associated with exposure to PFOA provide evidence that PFOA distributed unevenly between blood cells, and altered membrane fluidity and, in turn, altered downstream signaling pathways regulating platelets’ activation and aggregation. These findings could explain an association between PFAS exposure and CVD given that platelets play an important role in CVD both in the pathogenesis of atherosclerosis and in the development of acute thrombotic events [51].

This study was limited to cross-sectional analysis of serum PFAS levels at a certain period of time, which makes difficult to track the trends of PFAS levels in the study population or to pin-point significant exposure route(s). In addition, due to the cross-sectional design cause-and-effect associations have not been established for either MetS or any of individual components of MetS. Therefore, given the relatively long half-life of PFAS in the human body, long-term prospective studies (longitudinal studies) are urgently required to elucidate the putative causal relationships between PFAS and health effects. An imprecision in identifying MetS we also face is the lack of specific measurements for waist circumference and using self-reported height and weight that might cause inaccuracies in identify subjects with MetS. The study also lacked data on fasting blood glucose. Using more directly clinically relevant outcomes (e.g., elevated fasting glucose) would be preferable; however, because of using non-fasting blood to facilitate the recruitment process, this was not an option. 

Furthermore, while the survey data allowed us to control for a wide number of sociodemographic and lifestyle risk factors, due to complexity and lack of understanding of the interplay between exposure to PFAS and personal behavior, we cannot rule out the possibility of both positive and negative confounding, that are unaccounted for in our study, such as consumption of junk food. We have not studied interactions of multiple PFAS and other potential environmental toxicants, nor the assessment of combined exposure to multiple PFAS. 

Strengths of this study include examination of a large community residents, high quality of exposure measurements, detailed stratified analyses, adjusting a comprehensive profile of potentially confounding variables, and the ability to test for the robustness of our findings in various sensitivity analyses.

## 5. Conclusions

PFAS are suspected endocrine disruptors and human exposure is ubiquitous. In our large-scale cross-sectional study on young adult population, no associations were observed between legacy PFAS exposure and MetS, rather a negative association between PFOS and MetS. However, PFAS levels were significantly associated with some individual components of MetS and some showed protective associations, confirming results of previous researches and adding into the exciting evidence. Overall, further longitudinal follow-up studies on PFAS and their mixtures are warranted to clarify causal relationships.

## Figures and Tables

**Table 1:** Prevalence of metabolic syndrome (MetS) and individual components of MetS in the study population stratified by gender.

Prevalence of MetS	Total		Men		Women	
	*n*	%	*n*	%	*n*	%
MetS JIS * definition	743	4.68	507	6.57	236	2.89
**Prevalence of MetS components**						
**BMI ≥ 30 (obesity)**	1383	8.71	698	9.04	685	8.4
**Diabetics (modified parameters)**	135	0.85	74	0.96	61	0.75
HBA1c ≥6.1% or ≥43 mmol/mol	121	0.76	65	0.84	56	0.69
Medication, or self-reported diagnosis of type 2 diabetes	74	0.47	40	0.52	34	0.42
**Elevated TG**	1823	11.48	1382	18	441	5.41
TG ≥ 175 (mg/dL)	1820	11.46	1379	17.87	441	5.41
Drug treatment for elevated TG (fibrates)	3	0.02	3	0.04	0	0
**Reduced HDL-C**	2418	15.23	1071	14	1,347	16.51
HDL-C: < 40 mg/L (M); <50 mg/L (F)	2374	14.95	1041	13.49	1,333	16.34
Drug treatment for reduced HDL-C (statin)	65	0.41	45	0.58	20	0.25
**Elevated blood pressure**	5381	33.89	3722	48	1,659	20.34
Systolic ≥130 mmHg	4571	28.79	3331	43.16	1,240	15.2
Diastolic ≥85 mmHg	2484	15.65	1611	20.87	873	10.7
Antihypertensive drug treatment in a person with a history of hypertension	293	1.85	176	2.28	117	1.43

* The Joint Interim Statement on MetS definition (JIS).

**Table 2:** Distributions of the covariates in the study population (*n* = 15,876) according to metabolic syndrome (MetS) status for harmonised definition (JIS) stratified by gender.

Variables	Total					Males					Females				
	Non-MetS		MetS		*p*-Value	Non-MetS		MetS		*p*-Value	Non-MetS		MetS		*p*-Value
	Mean	SD	Mean	SD		Mean	SD	Mean	SD		Mean	SD	Mean	SD	
**Continuous covariates**															
Age	29.96	5.87	31.79	5.42	<0.001	29.75	5.88	31.6	5.48	<0.001	30.14	5.84	32.21	5.29	<0.001
Time-lag	14.72	5.46	15.85	5.28	<0.001	14.61	5.52	15.77	5.317	<0.001	14.82	5.41	16.02	5.21	<0.001
**Categorical covariates**															
	**Non-MetS**		**MetS**		***p*-Value**	**Non-MetS**		**MetS**		***p*-Value**	**Non-MetS**		**MetS**		***p*-Value**
	Freq.	%	Freq.	%		Freq.	%	Freq.	%		Freq.	%	Freq.	%	
**Clinical centers**															
Arzignano	3902	25.79	196	26.38	<0.001	1854	25.71	136	26.82	<0.001	2048	25.86	60	25.42	0.003
Legnago	3827	25.29	123	16.55		1828	25.35	81	15.98		1999	25.24	42	17.8	
San Bonifacio	3825	25.28	279	37.55		1766	24.49	194	38.26		2059	25.99	85	36.02	
Noventa Vicentina	3578	23.65	145	19.52		1763	24.45	96	18.93		1815	22.91	49	20.76	
**Alcohol consumption**															
NO	3540	23.4	224	30.15	<0.001	897	12.45	112	22.09	<0.001	2643	33.37	112	47.46	<0.001
< 3 per week	6168	40.78	278	37.42		2568	35.65	171	33.73		3600	45.45	107	45.34	
3–6 per week	3349	22.14	132	17.77		2112	29.32	121	23.87		1237	15.62	11	4.66	
> 7 per week	2068	13.67	109	14.67		1627	22.58	103	20.32		441	5.57	6	2.54	
**Smoking habbites**															
Non-smoker	8890	58.75	378	50.87	<0.001	3691	51.19	224	44.18	0.007	5199	65.63	154	65.25	0.83
Current smoker	4138	27.34	254	34.19		2399	33.27	199	39.25		1739	21.95	55	23.31	
Former smoker	2105	13.91	111	14.94		1121	15.55	84	16.57		984	12.42	27	11.44	
**Education**															
Elementary/Middle school	2213	14.62	217	29.21	<0.001	1205	16.71	152	29.98	<0.001	1008	12.72	65	27.54	<0.001
Highschool	9068	59.92	425	57.2		4583	63.56	292	57.59		4485	56.61	133	56.36	
University	3852	25.45	101	13.59		1423	19.73	63	12.43		2429	30.66	38	16.1	
**Number of deliveries**															
NO	12,148	80.33	634	85.33	<0.001	---					4937	62.4	127	53.81	<0.001
1	1275	8.43	38	5.11							1275	16.11	38	16.1	
2	1352	8.94	48	6.46							1352	17.09	48	20.34	
3+	348	2.3	23	3.1							348	4.4	23	9.75	
**Physical activity**															
Light	10,205	67.44	494	66.49	0.862	4424	61.35	326	64.3	0.123	5781	72.98	168	71.19	0.587
Moderate	2285	15.1	116	15.61		1078	14.95	81	15.98		1207	15.24	35	14.83	
Heavy	2642	17.46	133	17.9		1709	23.7	100	19.72		933	11.78	33	13.98	
**Country of birth**															
HDC	13,741	90.8	624	83.98	<0.001	6732	93.36	444	87.57	<0.001	7009	88.48	180	76.27	<0.001
HMPC	1392	9.2	119	16.02		479	6.64	63	12.43		913	11.52	56	23.73	
**Dietary factors ^1^**															
**Fruit/Vegetables**															
1Q	3972	26.27	230	30.96	0.002	2405	33.37	170	33.53	0.562	1567	19.8	60	25.42	0.07
2Q	4851	32.08	244	32.84		2390	33.16	177	34.91		2461	31.1	67	28.39	
3Q	3339	22.08	160	21.53		1327	18.41	95	18.74		2012	25.42	65	27.54	
4Q	2959	19.57	109	14.67		1085	15.05	65	12.82		1874	23.68	44	18.64	
**Milk/Yogurt**															
1Q	4618	30.52	231	31.09	0.167	2380	33.01	163	32.15	0.455	2238	28.25	68	28.81	0.418
2Q	3069	20.28	173	23.28		1412	19.58	114	22.49		1657	20.92	59	25	
3Q	6172	40.79	282	37.95		2829	39.24	192	37.87		3343	42.2	90	38.14	
4Q	1273	8.41	57	7.67		589	8.17	38	7.5		684	8.63	19	8.05	
**Cheese**															
1Q	4482	29.64	241	32.48	0.294	1907	26.47	149	29.45	0.498	2575	32.52	92	38.98	0.104
2Q	3879	25.65	181	24.39		1711	23.75	115	22.73		2168	27.38	66	27.97	
3Q	4035	26.68	182	24.53		2088	28.98	137	27.08		1947	24.59	45	19.07	
4Q	2727	18.03	138	18.6		1499	20.8	105	20.75		1228	15.51	33	13.98	
**Meat**															
1Q	6303	41.68	288	38.76	0.002	2283	31.69	167	32.94	0.162	4020	50.76	121	51.27	0.482
2Q	2624	17.35	101	13.59		1244	17.27	68	13.41		1380	17.43	33	13.98	
3Q	5387	35.62	307	41.32		3092	42.91	231	45.56		2295	28.98	76	32.2	
4Q	810	5.36	47	6.33		586	8.13	41	8.09		224	2.83	6	2.54	
**Sweets/Sweet beverages**															
1Q	5729	37.94	272	36.71	0.764	2868	39.86	186	36.69	0.403	2861	36.19	86	36.75	0.996
2Q	4377	28.98	210	28.34		1933	26.86	138	27.22		2444	30.92	72	30.77	
3Q	3492	23.12	180	24.29		1736	24.12	128	25.25		1756	22.21	52	22.22	
4Q	1503	9.95	79	10.66		659	9.16	55	10.85		844	10.68	24	10.26	
**Eggs**															
1Q	4261	28.17	289	38.9	<0.001	1900	26.37	186	36.69	<0.001	2361	29.8	103	43.64	<0.001
2Q	6287	41.56	274	36.88		2797	38.83	187	36.88		3490	44.05	87	36.86	
3Q	3066	20.27	110	14.8		1566	21.74	77	15.19		1500	18.93	33	13.98	
4Q	1512	10	70	9.42		941	13.06	57	11.24		571	7.21	13	5.51	
Fish															
1T	8984	59.37	460	61.91	0.382	4303	59.68	317	62.52	0.444	4681	59.09	143	60.59	0.892
2T	4027	26.61	184	24.76		1916	26.57	124	24.46		2111	26.65	60	25.42	
3T	2121	14.02	99	13.32		991	13.74	66	13.02		1130	14.26	33	13.98	
**Bread/Pasta/Cereals**															
1T	7040	46.56	335	45.09	0.543	3071	42.64	220	43.39	0.881	3969	50.13	115	48.73	0.603
2T	6308	41.72	312	41.99		3235	44.92	222	43.79		3073	38.82	90	38.14	
3T	1771	11.71	96	12.92		896	12.44	65	12.82		875	11.05	31	13.14	
**Salt consumption**															
Low	6418	42.41	265	35.67	0.001	2790	38.69	173	34.12	0.109	3628	45.8	92	38.98	0.117
Medium	7865	51.97	428	57.6		3981	55.21	298	58.78		3884	49.03	130	55.08	
High	850	5.62	50	6.73		440	6.1	36	7.1		410	5.18	14	5.93	

^1^ Dietary factors were categorized in quartiles (Q) of food intake and some for tertiles (T) of food intake.

**Table 3:** Generalized Additive Models (GAM) models for the association between perfluoroalkyl substances (PFAS) (ln µg/L) and metabolic syndrome (MetS), stratified by gender, adjusted by several covariates ^a^, using the continuous and PFAS quartiles.

PFAS	Total				Male				Female			
	OR	CI 95%		*p* Value	OR	CI 95%		*p* Value	OR	CI 95%		*p* Value
log-PFOA	1	0.93	1.07	0.997	0.98	0.9	1.07	0.639	1.03	0.91	1.17	0.649
I Q (0.35–13.5)	Reference				Reference				Reference			
II Q (13.6–35.3)	1	0.8	1.26	0.983	0.98	0.71	1.35	0.887	1.05	0.76	1.45	0.78
III Q (35.4–77.8)	1.04	0.82	1.32	0.749	1.02	0.75	1.39	0.906	1.05	0.72	1.54	0.804
IV Q (77.9–1400)	0.99	0.77	1.26	0.918	0.98	0.72	1.33	0.88	0.87	0.52	1.45	0.594
log-PFOS	**0.7** *	0.61	0.79	0	**0.68**	0.58	0.8	0	**0.71**	0.58	0.89	0.002
I Q (0.35–2.4)	Reference				Reference				Reference			
II Q (2.5–3.7)	**0.8**	0.64	0.99	0.043	0.9	0.66	1.21	0.473	**0.69**	0.5	0.96	0.029
III Q (3.8–5.6)	**0.78**	0.62	0.97	0.025	0.86	0.64	1.15	0.31	**0.67**	0.46	0.98	0.037
IV Q (5.7–142)	**0.55**	0.43	0.7	0	**0.58**	0.43	0.79	0	**0.5**	0.3	0.83	0.007
log-PFHxS	1.02	0.94	1.11	0.651	0.95	0.86	1.05	0.351	1.05	0.93	1.17	0.438
I Q (0.35–1.6)	Reference				Reference				Reference			
II Q (1.7–3.5)	0.97	0.76	1.24	0.814	1	0.69	1.46	0.98	1	0.72	1.39	0.999
III Q (3.6–7.8)	1.23	0.97	1.57	0.091	1.22	0.86	1.72	0.27	1.39	1.01	1.91	0.042
IV Q (7.9–127)	1.06	0.82	1.37	0.677	0.99	0.7	1.4	0.961	1.12	0.8	1.58	0.514
log-PFNA	0.81	0.65	1	0.05	0.76	0.59	0.97	0.03	0.92	0.61	1.39	0.695

^a^ All models are adjusted for age, gender, Time-lag between the beginning of the study and blood sampling Center where BP has been measured, Education, Number of deliveries, Physical activity, Country of birth, Diet, alcohol intake, and smoking status. * Bolded values indicate statistically significant result (*p* < 0.05).

**Table 4:** GAM models for the association between PFAS (ln µg/L) and metabolic syndrome components associated with one unit increase in log-PFAS, adjusted by several covariates ^a^.

PFAS	Elevated Blood Pressure			HDL-C			Elevated Triglycerides			Diabetes Status *			BMI ≥ 25			BMI ≥ 30		
	OR	CI 95%		OR	CI 95%		OR	CI 95%		OR	CI 95%		OR	CI 95%		OR	CI 95%	
**log-PFOA**																		
Model 1	**1.04** **	1.01	1.08	**0.94**	0.90	0.98	**1.08**	1.03	1.13	**0.74**	0.64	0.86	**0.95**	0.92	0.99	0.97	0.92	1.03
Model 2	**1.05**	1.01	1.08	**0.93**	0.89	0.97	**1.10**	1.05	1.16	**0.74**	0.63	0.85	**0.94**	0.91	0.97	0.97	0.91	1.02
**log-PFOS**																		
Model 1	1.04	0.98	1.11	**0.75**	0.70	0.81	**0.87**	0.79	0.95	**0.59**	0.45	0.78	**0.83**	0.78	0.89	**0.70**	0.63	0.77
Model 2	**1.10**	1.03	1.17	**0.79**	0.73	0.86	0.97	0.88	1.07	**0.63**	0.47	0.83	**0.86**	0.80	0.91	**0.73**	0.65	0.80
**log-PFHxS**																		
Model 1	**1.07**	1.03	1.12	0.96	0.91	1.01	**1.08**	1.02	1.14	0.90	0.74	1.08	1.02	0.98	1.07	1.03	0.97	1.10
Model 2	**1.07**	1.03	1.12	**0.94**	0.89	0.99	**1.09**	1.02	1.16	0.89	0.73	1.07	1.01	0.97	1.05	1.01	0.95	1.08
**log-PFNA**																		
Model 1	1.07	0.97	1.18	**0.79**	0.70	0.90	0.98	0.86	1.13	0.90	0.56	1.44	0.92	0.84	1.01	**0.85**	0.72	1.00
Model 2	1.10	0.99	1.21	**0.79**	0.69	0.90	1.06	0.92	1.23	0.95	0.58	1.53	0.93	0.84	1.03	**0.87**	0.74	1.03

^a^ Model 1 adjusted age, gender, Time-lag between the beginning of the study and blood sampling center where BP has been measured, Education, Number of deliveries, Physical activity, Country of birth, Diet, alcohol intake, and smoking status; Model 2 adjusted for Model 1 + other components of the metabolic syndrome. * Diabetes statues includes participants with HbA1c ≥ 6.1% (≥43 mmol/mol) or self-reported diabetes or drug treatment of elevated glucose (hypoglycemic medicines). ** The bold values are indicating the statistically significant associations (*p* < 0.05.)

## Data Availability

The data are not publicly available. The data presented in this study are available on request from the corresponding author.

## Supplementary Materials

The following are available online at https://www.mdpi.com/1660-4601/18/3/1194/s1, Table S1: Concentrations of perfluoroalkyl substances (PFAS; µg/L) in serum as per defined method detection limit for each compound; Table S2: Prevalence of metabolically healthy obesity (MHO) in the study population; Table S3: Distributions of serum PFAS concentrations (ng/mL) in the study population stratified by gender; Table S4: Number of components of the metabolic syndrome present among participants. Figure S1: Directed Acyclic Graph to identify set of a minimally sufficient set of variables to control for confounding.
