# Supplementary material for: Associations of Perfluoroalkyl Substances with Prevalence of Metabolic Syndrome in Highly Exposed Young Adult Community Residents—A Cross-Sectional Study in Veneto Region, Italy
Source: Int J Environ Res Public Health. 2021 Jan 29;18(3):1194. doi: 10.3390/ijerph18031194 (PMC7908308; doi:10.3390/ijerph18031194)
Supplement: Supplementary file 1 [file ijerph-18-01194-s001.pdf]

Table 1. Concentrations of perfluoroalkyl substances (PFAS; µg/L) in serum as per defined method detection limit for each compound.

Type of PFAS(carbon chain length)	Detection limit (LOD)	Quantification limit (LOQ)	Detected participants (≥LOQ)		Mean	Minimum	25th	50th	75th	Maximum
			Number	(%)						
PFBS (C4)	0.1	0.5	199	1.25	0.360	0.354	0.354	0.354	0.354	5.80
PFPeA (C5)	0.1	0.5	12	0.08	0.354	0.354	0.354	0.354	0.354	0.60
PFHxS (C6)	0.1	0.5	15,353	96.72	5.972	0.354	1.60	3.60	7.90	127.0
PFHxA (C6)	0.1	0.5	22	0.14	0.354	0.354	0.354	0.354	0.354	7.10
PFBA (C6)	0.1	0.5	273	1.72	0.362	0.354	0.354	0.354	0.354	23.90
PFHpA (C7)	0.1	0.5	99	0.62	0.359	0.354	0.354	0.354	0.354	15.10
PFOS (C8)	0.1	0.5	15,826	99.7	4.623	0.354	2.500	3.700	5.6	142.0
PFOA (C8)	0.1	0.5	15,851	99.86	59.76	0.354	13.7	35.90	79.0	1400.0
PFNA (C9)	0.1	0.5	7,915	49.86	0.535	0.354	0.354	0.354	0.600	39.70
PFDA (C10)	0.1	0.5	3,274	20.63	0.429	0.354	0.354	0.354	0.354	35.80
PFUnA (C11)	0.1	0.5	972	6.12	0.369	0.354	0.354	0.354	0.354	6.00
PFDoA (C12)	0.1	0.5	103	0.65	0.356	0.354	0.354	0.354	0.354	7.40

PFBS, perfluorobutanesulfonic acid; PFPeA, perfluoropentanoic acid; PFHxS, perfluorohexane sulfonate; PFHxA, perfluorohexanoic acid; PFBA, perfluorobutanoic acid; PFHpA, perfluoroheptanoic acid; PFOS, perfluorooctane sulfonate; PFOA, perfluorooctanoic acid; PFNA, perfluorononanoic acid; PFDA, perfluorodecanoic acid; PFUnA, perfluoroundecanoic acid, and PFDoA, perfluorododecanoic acid.

Figure 1S

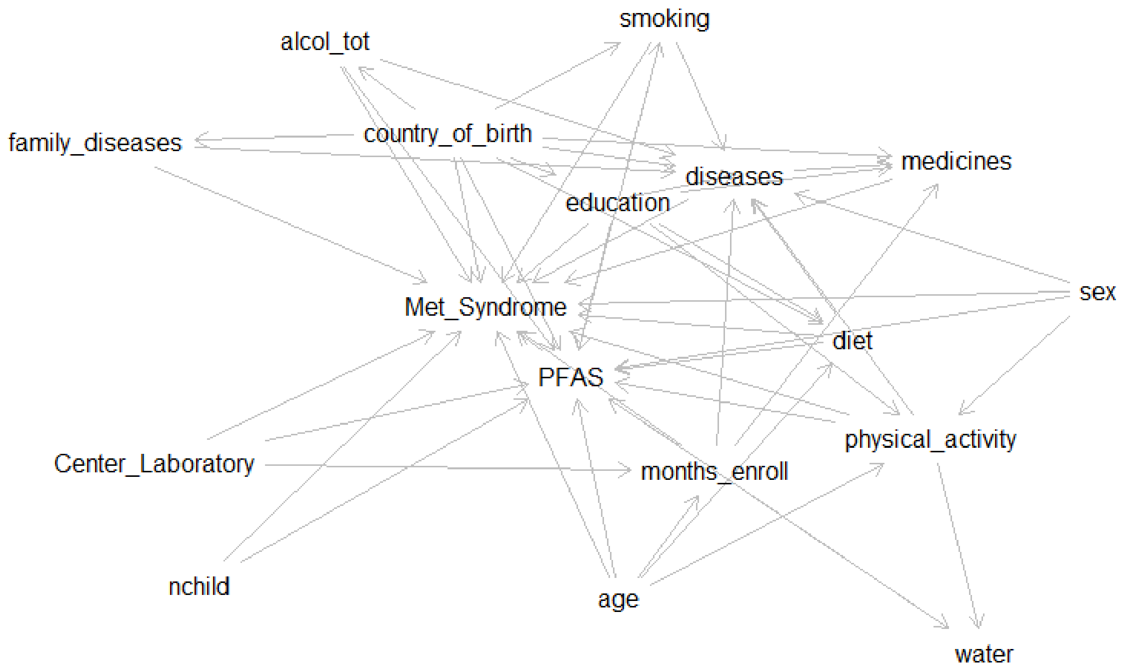

Table S2. Prevalence of metabolically healthy obesity (MHO) in the study population.

MHO	Overweight and obese (BMI>25)						obese(BMI>30)					
	Total		Males		Females		Total		Males		Females	
	Freq.	Percent	Freq.	Percent	Freq.	Percent	Freq.	Percent	Freq.	Percent	Freq.	Percent
NO	3,519	67.62	2,293	73.68	1,226	58.60	1,080	78.09	594	85.10	486	70.95
YES	1,685	32.38	819	26.32	866	41.40	303	21.91	104	14.90	199	29.05
Total	5,204	100	3,112	100	2,092	100	1,383	100	698	100	685	100

Table 3S. Distributions of serum PFAS concentrations (ng/mL) in the study population stratified by gender

Gender	PFAS	Participants who did not meet MetS criterias						Participants who met MetS criterias					
		Mean	min	p25	p50	p75	max	mean	min	p25	p50	p75	max
Total	PFOA	59.37	0.354	13.600	35.600	78.50	1253.3	67.66	0.700	16.80	41.40	87.80	1400.0
	PFOS	4.63	0.354	2.500	3.700	5.70	124.0	4.54	0.354	2.40	3.70	5.50	142.0
	PFHxS	5.89	0.354	1.600	3.500	7.80	77.1	7.54	0.354	2.00	4.70	9.50	127.0
	PFNA	0.53	0.354	0.354	0.354	0.60	8.8	0.58	0.354	0.35	0.50	0.60	39.7
Male	PFOA	83.92	0.354	25.100	58.500	115.30	1253.3	83.98	0.700	26.50	55.20	110.30	1400.0
	PFOS	5.75	0.354	3.300	4.800	7.00	88.6	5.25	0.600	2.80	4.20	6.10	142.0
	PFHxS	8.82	0.354	3.000	6.500	12.00	77.1	9.49	0.354	3.40	6.60	12.10	127.0
	PFNA	0.59	0.354	0.354	0.500	0.70	4.8	0.64	0.354	0.35	0.50	0.70	39.7
Female	PFOA	37.03	0.354	8.800	22.700	49.40	671.0	32.60	1.000	8.75	19.80	43.15	283.7
	PFOS	3.60	0.354	2.000	3.000	4.40	124.0	3.01	0.354	1.70	2.50	3.80	11.0
	PFHxS	3.23	0.354	1.100	2.200	4.30	41.3	3.37	0.354	1.20	2.40	4.45	17.0
	PFNA	0.48	0.354	0.354	0.354	0.50	8.8	0.45	0.354	0.35	0.354	0.50	2.2

Table 4S. Number of components of the metabolic syndrome present among participants

Number of MetS component	IDF (2005)		JIS definition*	
	**Freq.**	**Percent**	**Freq.**	**Percent**
0 criteria present (%)	10,672	67.22	8,098	51.01
1 criterion present (%)	1,702	10.72	5,348	33.69
2 criterion present (%)	2,220	13.98	1,687	10.63
3 criterion present (%)	965	6.08	563	3.55
4 criterion present (%)	305	1.92	171	1.08
5 criterion present (%)	12	0.08	9	0.06

***Alberti (2009)**
